# Fucosterol of Marine Macroalgae: Bioactivity, Safety and Toxicity on Organism

**DOI:** 10.3390/md19100545

**Published:** 2021-09-27

**Authors:** Maria Dyah Nur Meinita, Dicky Harwanto, Gabriel Tirtawijaya, Bertoka Fajar Surya Perwira Negara, Jae-Hak Sohn, Jin-Soo Kim, Jae-Suk Choi

**Affiliations:** 1 Seafood Research Center, Industry Academy Cooperation Foundation (IACF), Silla University, 606, Advanced Seafood Processing Complex, Wonyang-ro, Amnam-dong, Seo-gu, Busan 49277, Korea; dickyharwanto@lecturer.undip.ac.id (D.H.); ftrnd6@silla.ac.kr (G.T.); ftrnd12@silla.ac.kr (B.F.S.P.N.); jhsohn@silla.ac.kr (J.-H.S.); 2 Faculty of Fisheries and Marine Science, Jenderal Soedirman University, Purwokerto 53123, Indonesia; 3 Center for Maritime Bioscience Studies, Jenderal Soedirman University, Purwokerto 53123, Indonesia; 4 Faculty of Fisheries and Marine Science, Diponegoro University, Semarang 50275, Indonesia; 5 Department of Food Biotechnology, College of Medical and Life Sciences, Silla University, 140, Baegyang-daero 700 beon-gil, Sasang-gu, Busan 46958, Korea; 6 Department of Seafood and Aquaculture Science, Gyeongsang National University, 38 Cheong-daegukchi-gil, Tongyeong-si 53064, Korea

**Keywords:** fucosterol, seaweed, algae, toxicity, in vivo, in vitro

## Abstract

Fucosterol (24-ethylidene cholesterol) is a bioactive compound belonging to the sterol group that can be isolated from marine algae. Fucosterol of marine algae exhibits various biological activities including anti-osteoarthritic, anticancer, anti-inflammatory, anti-photoaging, immunomodulatory, hepatoprotective, anti-neurological, antioxidant, algicidal, anti-obesity, and antimicrobial. Numerous studies on fucosterol, mainly focusing on the quantification and characterization of the chemical structure, bioactivities, and health benefits of fucosterol, have been published. However, there is no comprehensive review on safety and toxicity levels of fucosterol of marine algae. This review aims to discuss the bioactivities, safety, and toxicity of fucosterol comprehensively, which is important for the application and development of fucosterol as a bioactive compound in nutraceutical and pharmaceutical industries. We used four online databases to search for literature on fucosterol published between 2002 and 2020. We identified, screened, selected, and analyzed the literature using the Preferred Reporting Items for Systematic Reviews and Meta-Analyses method and identified 43 studies for review. Despite the potential applications of fucosterol, we identified the need to fill certain related research gaps. Fucosterol exhibited low toxicity in animal cell lines, human cell lines, and animals. However, studies on the safety and toxicity of fucosterol at the clinical stage, which are required before fucosterol is developed for the industry, are lacking.

## 1. Introduction

Fucosterol is abundant and one of the dominant sterols in marine macroalgae [1]. The purest form of fucosterol and its potency were first identified and observed in the brown macroalga *Fucus vesiculosus* by Heilbron et al. [2]. Fucosterol is a stigmasterol bond isomer expressed by the empirical formula C_29_H_48_O. The fucosterol content in macroalgae ranges from 4 to 95% of the total phytosterol content [3]. Brown macroalgae contain higher levels of fucosterol than green and red macroalgae. The fucosterol content in brown macroalga *Ecklonia radiata* ranged between 312.0 µg/g dry weight in leaves and 378.1 µg/g dry weight in stipes (98.6 and 98.9% of total sterols, respectively) [4]. In the brown macroalgae *Himanthalia elongata*, *Undaria pinnatifida*, and *Laminaria ochroleuca*, fucosterol was observed predominantly in 83–97% of the total sterol content [5]. Fucosterol was also reported to be dominant in *Stephanocystis hakodatensis* (formerly *Cytoseira hakodatensis*) and *Sargassum fusiforme*, which contained 65.9% and 67% of fucosterol, respectively [6].

Brown macroalgae are widely used as food and herbal medicine in Southeast Asia and several European countries. In Europe, brown macroalgae have been used to treat goiter and obesity [7]. In East Asia, brown macroalgae from the genera *Laminaria*, *Undaria,* and *Sargassum* (formerly *Hizikia*) are widely consumed daily and used as herb medicine [8,9,10,11,12]. Hence, the bioactive properties of brown macroalgae have drawn the attention of researchers. Previous studies have investigated fucosterol properties and their potential bioactivities. The antioxidant effect of fucosterol was reported by Lee et al. [13]. Furthermore, Jung et al. [14] investigated the anti-inflammatory properties of fucosterol in LPS-stimulated conditions. Fucosterol has the potential to inhibit particulate-induced inflammation and oxidative stress in the alveolar cell line A549. Through regulation of the FoxO signaling pathway, fucosterol exhibits anti-obesity characteristics by suppressing adipogenesis in 3T3-L1 preadipocytes [15]. In addition, fucosterol protects human neuroblastoma cell line SH-SY5Y cells from amyloid-induced neurotoxicity [16] and affects human lung cancer cells by inducing apoptosis and cell cycle arrest and targeting the Raf/signaling mitogen-activated protein kinase/extracellular-signal-regulated kinase (MEK/ERK) pathway [17]. Based on these studies, fucosterol can potentially be developed for use in nutraceutical and pharmaceutical fields. However, before further developing fucosterol properties, information on the safety and toxicity of fucosterol is required to comprehend the optimum and sustainable benefits of fucosterol as a functional agent. This study reviews the current scientific literature regarding the bioactivity, safety, and toxicity of fucosterol extracted from marine macroalgae. In addition to the bioactivity of fucosterol, we investigated the safety and toxicity of fucosterol in various organisms, including bacteria/fungi, animal cell lines, human cell lines, and animals. Through this review article, we express our hope that the applications of fucosterol from marine algae can be further developed in the nutraceutical and pharmaceutical industries.

## 2. Results and Discussion

**Figure 1:**
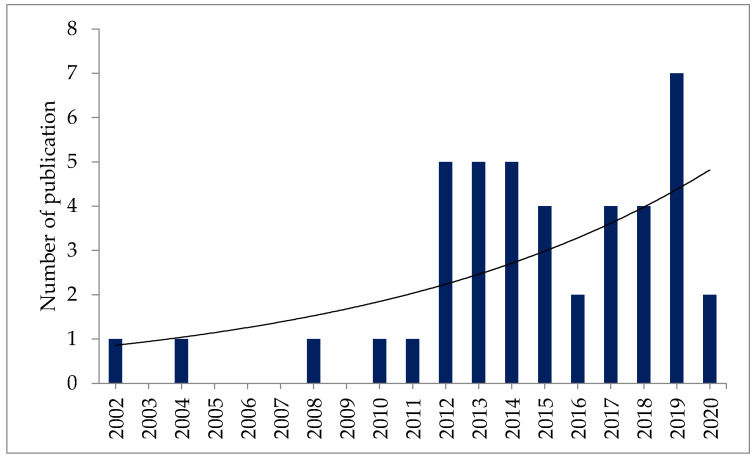
Number of publications on the safety and toxicity of fucosterol published in each year.

Studies on the bioactivity, safety, and toxicity levels of fucosterol from marine macroalgae conducted from 2002 to 2020 were reviewed, and an increasing trend was observed (Figure 1). This indicates that fucosterol has drawn additional research attention in recent years.

**Figure 2:**
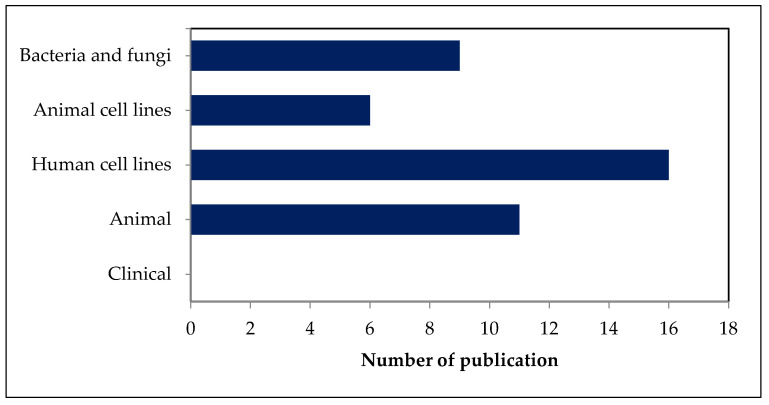
Numbers of publications on safety and toxicity of fucosterol categorized according to the treated organisms or cells.

Articles discussing the bioactivity, safety, and toxicity of fucosterol were categorized with respect to the various organisms or cells whose treatment they describe (Figure 2). Published studies on the safety and toxicity of fucosterol from marine algae have focused on its effects on bacteria and fungi (21%), animal cell lines (14%), human cell lines (38%), and animals (26%). The safety and toxicity of fucosterol have been studied both in vitro and in vivo. No clinical study of fucosterol has been conducted to date. Therefore, the investigation of the safety and toxicity of fucosterol at the clinical stage might be challenging.

**Figure 3:**
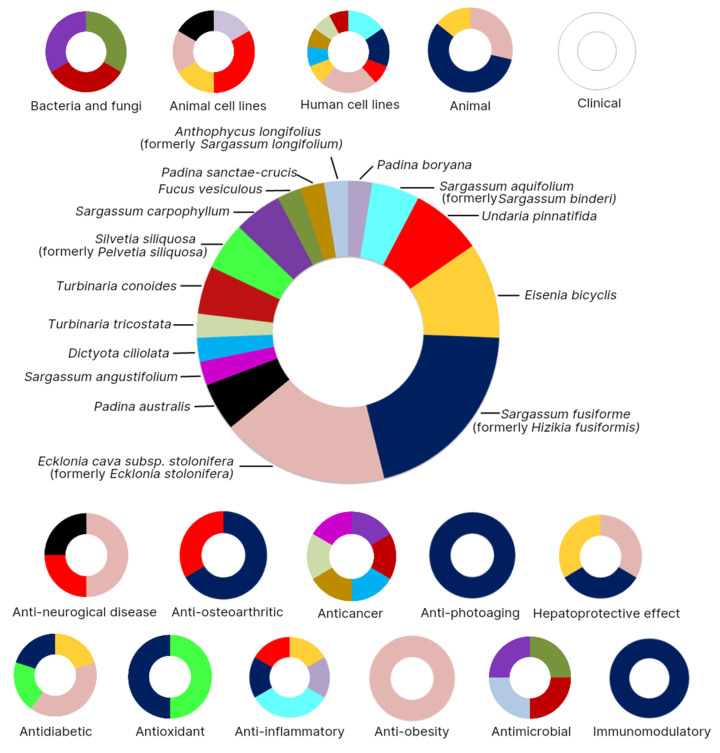
Numbers of publications on safety and toxicity of fucosterol classified according to sources of macroalgae.

The numbers of publications on the safety and toxicity of fucosterol from various sources of macroalgae are shown in Figure 3. Sixteen marine algae species belonging to Dictyotaceae, Sargassaceae, Alariaceae, Lessoniaceae, and Fucaceae families have been studied in relation to their fucosterol bioactivity, safety, and toxicity on bacteria, fungi, animal cells, human cells, and animals. Among the 16 marine algae, *Sargassum fusiforme* (formerly *Hizikia fusiformis*) has become the greatest marine macroalgal source for fucosterol that has been studied. Over the last 10 years, studies on the bioactivities and nutritional and pharmacological properties of *S. fusiforme* have increased steadily. Most of the studies on the bioactivity of *S. fusiforme* have focused on its antioxidant (15.09%), anticancer and antitumor (15.09%), anti-inflammatory (11.32%), photoprotective (11.32%), and neuroprotective (11.32%) properties [18]. Figure 3 shows that the study of fucosterol in *S. fusiforme* has focused on its antioxidant, anti-osteoarthritic, anti-inflammatory, anti-photoaging, antidiabetic, hepatoprotective, and algicidal effects. *Ecklonia cava* subsp. *stolonifera* (formerly *Ecklonia stolonifera*) is the second most frequently reported macroalgal species that has been studied for its fucosterol content. *Ecklonia* species have been known as potential source of bioactive compounds [19]. Studies on the safety and toxicity levels of fucosterol obtained from *Ecklonia stolonifera* were mostly conducted on animal cell lines, human cell lines, and animals. Fucosterol from *Ecklonia stolonifera* has been studied for its antidiabetic, anti-obesity, anti-neurological, and hepatoprotective effects. The safety and toxicity levels of fucosterol obtained from *Ecklonia cava* subsp. *stolonifera* were mostly studied in animals.

### 2.1. Characteristics and Structure of Fucosterol

Generally, fucosterol could be obtained by extracting dry powder of macroalgae using MeOH, EtOH, or *n*-hexane solvents [13,20,21,22,23,24]. The extracts were then partitioned via solvent fractionation [13,25]. After solvent dissolution under reduced pressure, the organic extracts were fractionated using silica gel column chromatography with a mixture of solvents of increasing polarity [14,22,23,26]. The fraction was eluted using a solvent to remove fatty acids, which were then analyzed further [26].

**Figure 4:**
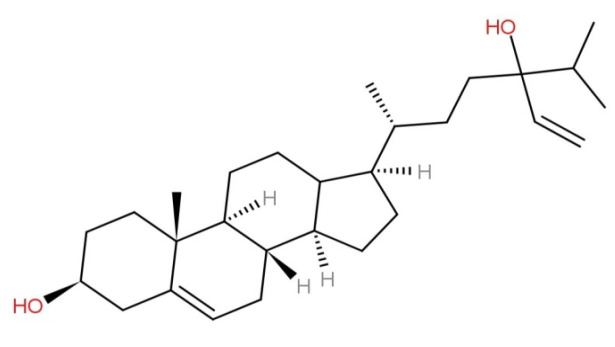
Chemical structure of fucosterol.

Currently, fucosterol analysis and identification are carried out using physical properties and spectroscopic methods, including ^1^H-NMR and ^13^C-NMR, as well as via comparison with published data, and thin-layer chromatography (TLC) analysis [20]. According to the molecular formula of fucosterol, one hydroxy group must be attached to C-28 in an *R* or *S* configuration. An olefin proton signal and two sets of two olefin signals indicate the presence of a tri-substituted double in the fucosterol side chain [22]. Infrared (IR) absorption peaks of fucosterol at 3400 and 1600 cm^−1^ were attributed to the hydroxyl and olefin groups, respectively. Fucosterol derivatives, namely 24*R*,28*R*- and 24*S*,28*R*-epoxy-24-ethylcholesterol, and 24*R*-saryngosterol have also been used in octadecyl silica gel (ODS) column chromatography [21]. The structure of fucosterol is shown in Figure 4.

### 2.2. Bioactivity of Fucosterol

**Figure 5:**
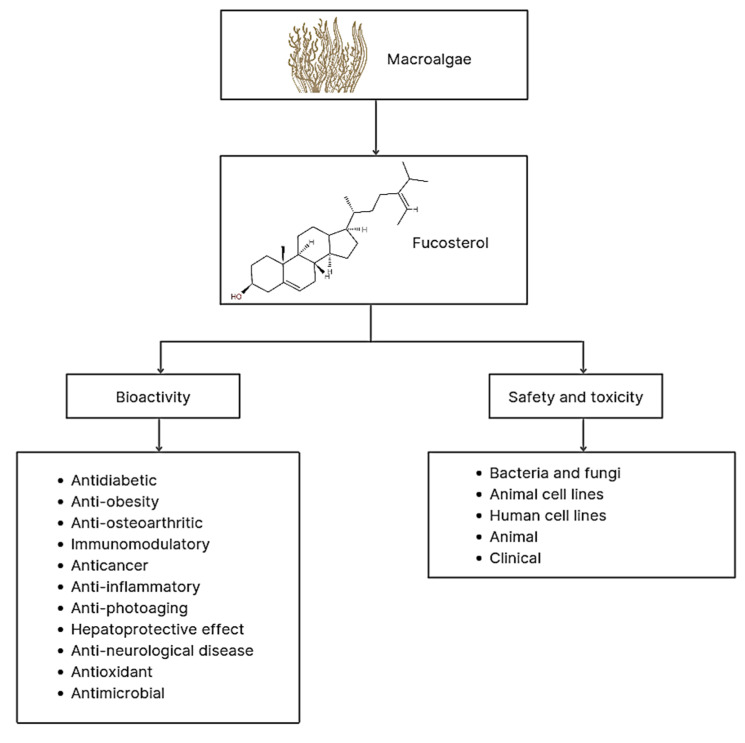
Bioactivities, safety, and toxicity levels of fucosterol derived from macroalgae.

Fucosterol of marine macroalgae exhibited antidiabetic, anti-obesity, anti-osteoarthritic, immunomodulatory, anticancer, anti-inflammatory, anti-photoaging, hepatoprotective, anti-neurological, antioxidant, and antimicrobial activities (Figure 5).

#### 2.2.1. Antidiabetic Activity

Diabetes is a chronic disease that occurs when the pancreas does not produce enough insulin or when the body cannot use insulin effectively. One of the antidiabetic effects of fucosterol obtained from *Eisenia bicyclis* and *Ecklonia cava* subsp. *stolonifera* is characterized by inhibition of enzymes, such as rat lens aldose reductase (RLAR), human recombinant aldose reductase (HRAR), α -glucosidase, and PTP1B. The docking simulations clearly demonstrated negative binding energy for fucosterol (−8.2 kcal mol^−1^ for RLAR and −8.5 kcal mol^−1^ for HRAR), implying a higher affinity and stronger binding competence for the active site of the enzyme [20]. These results were confirmed by Jung et al. [27], who stated that *Ecklonia stolonifera*-derived fucosterol reduced insulin resistance by decreasing PTP1B expression and activating insulin signaling pathways. Fucosterol from *Sargassum fusiforme* also showed strong PTP1B inhibition at low concentrations [28]. Streptozotocin-induced diabetic rats treated with fucosterol from *Pelvetia siliquosa* via an orally administered dose of 30 mg/kg showed a decrease in serum glucose levels and inhibition of its accumulation. However, orally administered doses of 300 mg/kg are required for epinephrine-induced diabetic rats to inhibit blood glucose levels and degrade glycogen [29]. These findings illustrate that fucosterol extracts from *Eisenia bicyclis*, *Ecklonia cava* subsp. *stolonifera*, *Sargassum fusiforme*, and *Silvetia siliquosa* have antidiabetic potential that can be developed in the future.

#### 2.2.2. Anti-Obesity Activity

In addition to being antidiabetic, fucosterol also exhibits anti-adipogenic or anti-obesity properties. Adipocytes have important cardiovascular-related roles; therefore, understanding their development and regulation is important for treating obesity and related diseases. Obesity is associated with hypercholesterolemia, diabetes, and other chronic diseases. Hypercholesterolemia is associated with a higher incidence of liver damage, especially non-alcoholic fatty liver disease (NAFLD) [30]. As reported by Jung et al. [25], following treatment with fucosterol extracted from *Ecklonia cava* subsp. *stolonifera*, lipid accumulation in 3T3-L1 pre-adipocytes decreased because the expression levels of adipocyte markers, proteins peroxisome proliferator-activated receptor (PPAR), and CCAAT/enhancer-binding protein (C/EBP), decreased [25]. Similar results were reported by Lee et al. [15], who found that fucosterol of *Ecklonia cava* subsp. *stolonifera* inhibits adipogenesis of 3T3-L1 preadipocytes via modulation of the FoxO signaling pathway. 

#### 2.2.3. Anti-Osteoarthritis Activity

The anti-osteoarthritis activity of fucosterol has been demonstrated both in vitro and in vivo [21,31,32]. Anti-osteoarthritis is a biological activity characterized by the ability of a compound to reduce or prevent bone disease. *Sargassum fusiforme*-derived fucosterol has been shown to increase proliferative activity in osteosarcoma MG63 cells for the treatment of bone-absorbing metabolic bone diseases, including osteoporosis and periodontitis [21]. Similar results were observed in ovariectomized rat osteoporosis and fucosterol of *Sargassum fusiforme* triggered bone regeneration and activation of bone formation [31], and Bang et al. [32] tested fucosterol in vitro and reported that fucosterol from *Undaria pinnatifida* inhibited osteoclast differentiation. Through this biological activity, fucosterol may play an important role in preventing osteoporosis and may be useful as a supplement.

#### 2.2.4. Immunomodulatory Activity

In immunomodulation, regulating many immune cells through signaling molecules to enhance the immune system, is necessary. Based on previous studies, several marine metabolites have been reported to have regulatory effects on the immune system [33]. Fucosterol is one of the compounds that is regarded as a candidate immunomodulator, as reported by Park et al. [34] in vitro and in vivo; fucosterol from *Sargassum fusiforme* can increase the secretion of tumor necrosis factor alpha (TNF-α), NO production, and phagocytosis activity. Generally, fucosterol has the potential to regulate immune function and may offer positive therapeutic effects in immune system diseases.

#### 2.2.5. Anticancer Properties

Certain studies about the anticancer properties of fuscosterol extracted from marine macroalgae, such as *Sargassum carpophyllum*, *Turbinaria conoides*, *Dictyota ciliolata*, and *Padina sanctae-crucis*, have been published [21,35,36,37,38,39]. Based on the study by Jiang et al. [39], commercial fucosterols exhibit anticancer activity by inhibiting the PI3K/Akt/mTOR signaling pathway in cervical cancer cell lines. In addition, fucosterol from *Sargassum fusiforme* has been observed to slow the progression of human ovarian cancer [35] and inhibit the proliferation of osteosarcoma-derived cell MG63 [21]. Tang et al. [22] isolated steroids from *Sargassum carpophyllum* using activity-guided fractionation to determine the effect of fucosterol and other active substances on cancer cell lines. This activity is indicated by IC_50_, which is the concentration that results in 50% inhibition of cell growth [37]. They found that fucosterol has an IC_50_ value of 7.8 µg/mL against HL-60 cancer cells. A compound can exhibit one of three types of cytotoxicity: (1) potential cytotoxicity, if IC_50_ < 100 µg/mL, (2) moderate cytotoxicity, if 100 g/mL < IC_50_ < 1000 µg/mL, and it can be (3) non-toxic, if IC_50_ > 1000 µg/mL. Agents from a group of potentially cytotoxic compounds can be utilized as anticancer drugs, while moderately cytotoxic compounds can be used for chemoprevention to stop cancer cell growth [40]. According to the National Cancer Institute (NCI), a compound is classified as having anticancer properties if its IC_50_ is <20 µg/mL. Published research studies prove that fucosterol as a metabolite compound in macroalgae has anticancer properties; however, further clinical studies are required. 

#### 2.2.6. Anti-Inflammatory Activity

Inflammation is a biological response to noxious stimuli. It is a protective response involving immune cells such as macrophages, blood vessels, molecular mediators such as NO, pro-inflammatory cytokines (TNF-α, interleukin-1β (IL-1β), and interleukin-6 (IL-6)), and prostaglandins [41]. Previous studies have identified anti-inflammatory compounds in macroalgae. Fucosterol derived from the methanolic extract of the brown alga *Eisenia bicyclis* and *Undaria pinnatifida* exhibits anti-inflammatory properties by suppressing the production of cyclooxygenase-2 (COX-2) and inducible nitric oxide synthase (iNOS) in LPS-stimulated RAW 264.7 macrophages [14,42]. Fucosterol also inhibited t-BHP-induced ROS production and suppressed iNOS and COX-2 expression. Furthermore, *E. bicyclis* has strong anti-inflammatory properties with the potential to inhibit NO and ROS production, as well as the NF-κB pathway [14]. Fucosterol from *Padina boryana* was reported to have anti-inflammatory properties via the regulation of NF-κB/MAPK and involvement of Nrf2/HO-1 pathways in PM-induced inflammation/oxidative stress in RAW 264.7 macrophage cells [43]. Furthermore, fucosterol extracted from *Sargassum aquifolium* (formerly *Sargassum binderi*) decreased ROS levels in PM-induced HaCaT cells and human lung epithelial cells [15,44]. Another study also showed inhibition of hypoxia-inducible factor through the PI3K/Akt pathway in keratinocytes (HaCaT) cells induced by cobalt chloride (CoCl_2_). Based on the research conducted, fucosterol from brown macroalgae has the potential to be developed further in the pharmacological field.

#### 2.2.7. Anti-Photoaging Effect

Photoaging is a result of chronic ultraviolet irradiation, which is one of the most harmful environmental factors affecting the skin. Recently, studies on marine compounds as safe anti-photoaging alternatives have been published by Hwang et al. [23]. They reported that fucosterol obtained from the brown alga *Sargassum fusiforme* was observed to have anti-photoaging properties. Fucosterol therapy decreased ultraviolet B (UVB)-induced production of matrix metalloproteinase-1 (MMP-1), IL-6, p-c-Jun, and p-c-Fos and increased type I and transforming growth factor-1 (TGF-1) procollagen expression in normal human dermal fibroblast cells. In addition, fucosterol derived from *Sargassum fusiforme* was observed to regulate the expression of MMPs and type-I pro-collagen in UV-irradiated HaCaT cells by modulating, microtubule associated protein kinase (MAPK) [45]. These findings suggest that fucosterol extracted from *Sargassum fusiforme* is a potential candidate for the prevention and treatment of skin aging.

#### 2.2.8. Hepatoprotective Effect

The liver is the largest organ in the abdominal cavity and performs critical physiological functions. Acute liver failure is caused by liver injury, which is mainly caused by viral infections, drugs, food additives, alcohol, or radioactivity [46]. Fucosterol has a potent hepatoprotective effect by increasing GSH levels and decreasing ROS production, thereby preventing liver damage and increasing liver enzyme levels alanine aminotransferase/ aspartate aminotransferase (ALT/AST) [47]. Fucosterol is a dual-LXR agonist that regulates the expression of key genes involved in cholesterol homeostasis in several cell lines without causing triglyceride accumulation in the liver [48,49]. In addition, a study by Mo et al. [50] showed that fucosterol can relieve acute liver injury induced by ConA by inhibiting P38 MAPK/PPARγ/NF-κB signaling, suggesting that fucosterol is a promising potential therapeutic agent for acute liver injury.

#### 2.2.9. Anti-Neurological Disease

Neurological disorders are diseases of the nervous system, such as brain tumors, epilepsy, Parkinson’s disease, stroke, and Alzheimer’s disease (AD) [51]. In a study conducted by Yoon et al. [24], fucosterol extracted from *Ecklonia cava* subsp. *stolonifera* showed the presence of cholinesterase inhibitors against AchE and BchE. BChE was inhibited by fucosterol and 24-hydroperoxy 24-vinylcholesterol, with IC_50_ values of 421.72 ± 1.43 and 176.46 ± 2.51 µM, respectively. The effect of the compound on amyloid-induced neurotoxicity can be used to determine the potential of the compound as an anti-AD [16,52]. Consequently, *Padina australis*-derived fucosterol reduced intracellular amyloid levels and increased neuroglobin mRNA expression in amyloid-induced SH-SY5Y cells [16]. Fucosterol extracted from *Ecklonia cava* subsp. *stolonifera* co-infusion attenuated cognitive impairment-induced sAβ_1-42_ in aging mice via the downregulation of GRP78 expression [52]. Furthermore, the anti-AD properties of fucosterol from macroalgae have also been reported by Jung et al. [53] and Wong et al. [54]. Research on effects of fucosterol against Parkinson’s disease has been described by Paudel et al. [55], and it was found to exhibit a mild dopamine D4 antagonist effect by inhibiting the dopamine agonist effect by 32% at 100 µM. Furthermore, fucosterol extracted from *Sargassum fusiforme* has also been reported to inhibit epilepsy and act as an antidepressant. The group treated with 20 mg/kg fucosterol showed a significant increase in the hippocampal brain-derived neurotrophic factor (BDNF) levels (*p* < 0.05). Published studies show that fucosterol from marine algae can be an alternative compound for the treatment of neurological diseases.

#### 2.2.10. Antioxidant Activity

Excessive ROS formation can trigger oxidative stress, which causes cell damage and changes cell functions. Antioxidants are required to maintain a balance and prevent negative effects from excessive ROS formation. Based on the literature reviewed, macroalgae showed antioxidant activity, which can be useful for preventing excessive ROS formation. Fucosterol from *Sargassum fusiforme* exhibited antioxidant properties by downregulating serum transaminase activity in CCl_4_-intoxicated rats. Sequentially, sGOT and sGPT activity decreased by 25.57% and 63.16%, respectively. In addition, fucosterol treatment of CCl4-intoxicated rats also increased hepatic cytosolic SOD, catalase, and GSH-px [13]. Oktaviani et al. [56] reported that fucosterol from *Hizikia fusiformis* prolonged the lifespan of *Caenorhabditis elegans* (Nematoda). Based on these studies, fucosterols extracted from macroalgae are potential candidates for antioxidants that can be used in functional foods and medicines.

#### 2.2.11. Antimicrobial Activity

Antimicrobial activities can be defined as the process of inhibiting or destroying the growth of microorganisms, especially pathogenic microorganisms. The antimicrobial properties, including antibacterial and antifungal properties of marine macroalgae, are associated with various groups of bioactive lipids, such as fucosterol. The results of the study by Tyskiewicz et al. [57] showed that fucosterol from *Fucus vesiculosus* at a concentration of 1.0% completely inhibited the germination of macroconidia in *Fusarium culmorum* (Fungi, Ascomycota). Furthermore, when macroconidia were exposed to low doses of fucosterol (0.05–0.2%), their growth was inhibited, and structural degradation occurred. Furthermore, fucosterol from *Sargassum carpophyllum* cultured with *Pyricularia oryzae* (Fungi, Ascomycota) mycelia caused abnormal morphological changes [22]. Previous studies confirmed the antibacterial and antifungal activity of 3,6,17-trihydroxy-stigmasta-4,7,24(28)-triene, fucosterol, and 14,15,18,20-diepoxyturbinarin compounds from *Turbinaria conoides*, with MICs ranging from 2 to 16 µg/mL, against *Staphylococcus aureus*, *S. epidermidis*, *Escherichia coli*, *Pseudomonas aeruginosa*, *Aspergillus niger*, and *Candida albicans* [58]. The antibacterial properties of *Sargassum longifolium* fucosterol were also tested against the human pathogen *Vibrio parahaemolyticus* and the fish pathogens *V. vulnificus*, *V. harveyii*, and *Aeromonas hydrophililla*. Interestingly, among these bacteria, only *P. fluorescens* was not susceptible to the effect of fucosterol [59]. Overall, fucosterol could potentially be a strong and promising antimicrobial agent.

### 2.3. Safety and Toxicity of Fucosterol in Bacteria and Fungi

Several studies on fucosterol in bacteria and fungi have been published [22,57,58]. From these articles, data extraction was performed, as shown in Table 1.

According to Tang et al. [58], fucosterol isolated from *Sargassum carpophyllum* showed low toxicity, with IC_50_ = 250 µg/mL, and was able to induce morphological changes in *Pyricularia oryzae*. Furthermore, fucosterol extract from *Turbinaria conoides* was used to test the level of growth inhibition in bacteria (*S. aureus*, *S. epidermidis*, *E. coli*, and *P. Aeruginosa*) and fungi (*C. albicans* and *A. niger*). In the tested bacteria, the MIC values ranged from 8 to 16 µg/mL, which indicated that fucosterol was able to inhibit the growth of the tested bacteria well. In addition, fucosterol showed the highest growth inhibition in *C. albicans*, with MIC = 8 µg/mL. Furthermore, research by Tyskiewicz et al. [57] showed that at a concentration of 1.0% fucosterol was able to optimally inhibit the growth of *F. culmorum* macroconidia. Moreover, macroconidia showed shorter length and structural degradation at lower fucosterol concentrations (0.05–0.2%).

From these studies, we conclude that fucosterol can potentially be developed as a new agent for combating the problem of infection due to bacteria and fungi that are pathogenic because of its excellent biological activity as an inhibitor of bacteria and fungi. Based on our literature review, only the genera *Turbinaria* and *Sargassum* have been studied for the treatment of pathogenic bacteria and fungi. No other genera have been reported with respect to their safety and toxicity in bacteria and fungi. Further research on other bacteria and fungi species is required to comprehensively elucidate the safety and toxicity of fucosterol in bacteria and fungi.

### 2.4. Safety and Toxicity of Fucosterol in Cell Lines

Several studies have demonstrated safety and toxicity in human cell lines [17,27,28,35,36,37,38,47,53,60,61,62] and animal cell lines [15,42,43,45,54,55]. The safety and toxicity of fucosterol in human and animal cell lines are summarized in Table 2.

In a study involving the use of commercial fucosterol for the treatment of RAW 264.7 macrophage cell line stimulated by particulate matter (PM), Jayawardena et al. [43] demonstrated the inhibition of NO production levels by observing inflammatory mediators, such as iNOS, COX-2, and pro-inflammatory cytokines (i.e., IL-6, interleukin-1β (IL-1β), and tumor necrosis factor-α (TNF-α)), including prostaglandin E2 (PGE2)). Furthermore, the effect of fucosterol was amplified by the decreased expression of mitogen-activated protein kinase (MAPK) and NF-κB signaling pathway molecules and ROS regulation. Fernando et al. [44] showed that increasing concentrations (>100 µg/mL) of Chinese fine dust PM (CPM) in A549 cells significantly increased ROS levels and caused cell death. CPM-induced A549 cells treated with fucosterol of *Sargassum aquifolium* at a concentration of 3.125–50 µg/mL caused an increase in cell viability of up to 94.98 ± 1.26%, and the IC_50_ value was estimated to be 21.74 ± 0.67 µg/mL [60]. In addition, *Sargassum aquifolium*-derived fucosterol was also reported to increase cell viability in HaCaT and HDF cells and yielded non-toxic results.

Another study reported that the addition of fucosterol isolated from *Sargassum fusiforme* in HaCaT cells induced by 500 µM CoCl_2_ increased cell viability up to 56% at a concentration of 10 µM [61]. Furthermore, as reported by Choi et al. [47], there was no effect on HepG2 cells after treatment with the crude extract of fucosterol up to a concentration of 100 µM. However, in HepG2 cells induced by t-BHP and tacrine, fucosterol showed low toxicity and increased cell viability. In addition, LPS-induced RAW 264.7 macrophages treated with fucosterol of *Undaria pinnatifida* showed an unclear toxic effect because the 3-(4, 5-dimethyl thiazole-2-yl)-2, 5-diphenyltetrazolium bromide MTT test results showed that fucosterol did not affect cell viability at a concentration of 10–50 µM [42]. These findings are similar to those of Wong et al. [54] at concentrations ranging from 12 to 192 µM, fucosterol of *Padina australis* did not have a significant cytotoxic effect on C8-B4 cells when compared with control (untreated cells) (approximately 100% cell viability). 

Research conducted by Paudel et al. [55] on activity of fucosterol from *Undaria pinnatifida* and *Eisenia bicyclis* reported that there was no visible effect of the crude fucosterol extract on MAO-A and MAO-B (half-maximal inhibitory concentration (IC_50_) > 500 µM). MAO is a catecholamine-degrading enzyme with a long-standing therapeutic profile; MAO-A and MAO-B are isoenzymes. In addition, the results of the functional assay showed that fucosterol did not show agonist activity at any of the receptors tested. Moreover, the crude extract of fucosterol can potentially inhibit PTP1B and α-glucosidase with an IC_50_ value of 50.58 ± 1.86 µM [28], and BACE1 with an IC_50_ value of 64.12 ± 1.0 µM [53] without causing side effects.

In a report by Lee et al. [15], treatment with fucosterol from *Ecklonia cava* subsp. *stolonifera* of 3T3-L1 preadipocytes had no effect on inhibiting cell proliferation up to a concentration of 50 µM [15]. This finding is complemented by previous investigations that stated that the survival of HepG2 cells was not affected up to a concentration of 100 µM *Ecklonia cava* subsp. *stolonifera*-derived fucosterol for 24 h. However, cell survival was reduced to 48 h at a concentration of 200 µM. Based on these results, they recommended that additional in vitro studies on the anti-diabetic activity of fucosterol be carried out using non-toxic concentrations of 50, 25, and 12.5 µM [27].

The cytotoxicity of fucosterol in various human cancer cells has been widely published [17,62,64,65]. According to Mao et al. [17], commercial fucosterol inhibited the growth of all human lung cancer cells tested with IC_50_ values ranging from 15 to 60 µM. However, interestingly, fucosterol showed low toxicity in all normal cells with IC_50_ > 100 µM, which indicated that fucosterol can selectively inhibit lung cancer cell growth, induce cell cycle arrest, and target the Raf/MEK/ERK signaling pathway. Other studies have shown that commercial fucosterol reduces cell viability and enhances the cytotoxic effect of 5-Fu in HT29 cancer cells without affecting normal colon fibroblasts (CCD-18Co). Studies on the toxicity of fucosterol on HL-60 [63]; ES2 and OV90 [35], HT-29, Caco-2, and T47D [38]; KB, Hep-2, MCF-7, and SiHa [37] yielded low to no toxicity results in DLA cells [36].

In previous studies, the safety and toxicity of fucosterol in human and animal cell lines have been extensively investigated. Most of the studies focused only on the brown seaweeds of genera *Sargassum*, *Undaria*, *Turbinaria*, and *Ecklonia*. Based on the literature reviewed, other genera, such as *Ulva* and *Enteromorpha*, have not been tested in cell lines. Fucosterol from these genera has been reported to have biological activity, but its safety and toxicity levels in cell lines have not been reported. Therefore, the mechanisms of other genera in cell lines should be investigated further.

### 2.5. Safety and Toxicity of Fucosterol in Animals

Several studies on the safety and toxicity of fucosterol isolated from marine macroalgae in animals have been published. The brown seaweed *Ecklonia cava* subsp. *stolonifera* [56,57] and *Hizikia fusiformis* or *Sargassum fusiformis* [31,34,56,66] were the most frequently discussed. A summary of the safety and toxicity of fucosterol in animals is presented in Table 3.

The research of Mo et al. [50], complements the data about the anti-diabetic and anti-obesity properties of fucosterol that inhibits necrosis and apoptosis in a process mediated by PPARγ activation and inhibition of NF-B, which reduces inflammatory factors. Fucosterol also inhibits apoptosis and autophagy by upregulating Bcl-2 via PPARγ, thereby decreasing functional Bax and Beclin-1. Park et al. [34] reported that administration of 200 mg/kg body weight to mice increased splenocyte proliferation and NO production without cytotoxicity. Based on the research by Oktaviani et al. [56], fucosterol from *Sargassum fusiforme* showed low toxicity because it significantly affected the survival of *C. elegans* (1.54-fold and 1.23-fold increase), at a concentration of 0.05 mg/mL.

Some studies have been conducted to test the effect of fucosterol on the nervous system. Oh et al. [52] reported no toxicological response induced by the administration of fucosterol derived from *Ecklonia stolonifera* when it was injected at 10 mol/h into the dorsal hippocampus for four weeks. After training of aging rats, there was an increase in latency to reach the platform. Furthermore, Zhen et al. [66] showed no neurotoxic effect at the same dose levels administered after 0.5 and 4 h. Conversely, fucosterol from *Sargassum fusiforme*, showed no neurotoxic activity at the doses used in the forced or tail suspension tests (10, 20, 30, and 40 mg/kg). Lee et al. [31] found that all doses of fucosterol from *Sargassum fusiforme* had no toxic effects in OVX mice. The results of the study showed that fucosterol treatment significantly improved the loss of bone density caused by ovariectomy. Furthermore, the research conducted by Choi et al. [47] showed that there were no deaths or gross appearance abnormalities, and no fucosterol-induced abnormal behavioral changes, seizures, or death over 24 h due to *Ecklonia cava* subsp. *stolonifera* and *Eisenia bicyclis*. However, pretreatment with fucosterol at doses of 25, 50, and 100 mg/kg body weight markedly attenuated this cytotoxic effect of tacrine. In addition, the hepatoprotective effect of fucosterol at the highest dose (100 mg/kg body weight) resulted in serum ALT levels similar to those of the control group, suggesting that fucosterol has the potential to reduce tacrine-induced hepatotoxicity. The published results of fucosterol studies indicate that the number of in vivo tests involving algal metabolites is very limited. The experimental model that has been used thus far has focused on mice. Therefore, we propose that future research should focus on determining the full in vivo potency of fucosterol.

## 3. Materials and Methods

### 3.1. Literature Search

The preferred reporting items for systematic reviews and meta-analyses (PRISMA) method [67] were used for the collection, identification, screening, selection, and analysis of the studies reviewed. A literature search was performed using four databases: PubMed, Science Direct, Wiley, and Web of Science. The search criteria included scientific articles on fucosterol published between 2002 and 2020. The keywords used in the literature search were “fucosterol” and “bioactivites OR “biological activities” OR “safety” OR “toxicity” OR “characteristics” OR “structure” OR “cell lines” OR “microalgae” OR “macroalgae” OR “plant” OR “bacteria” OR “fungi” OR “invertebrates” OR “animals” OR “human.” The total number of articles found was 1251, which, upon further screening by checking the title and keywords and removing similar articles, was decreased to 621.

### 3.2. Selection Criteria

**Figure 6:**
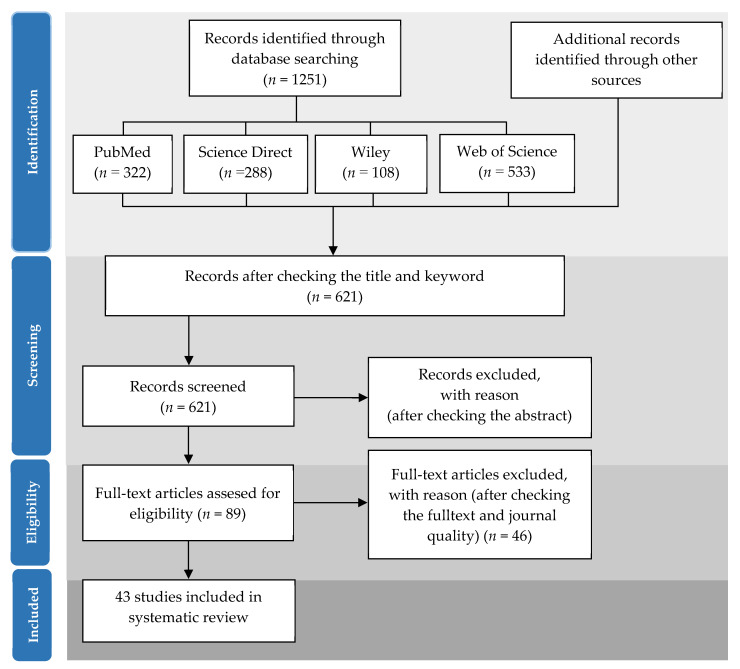
Summarized search method based on PRISMA.

In the second screening stage, a total of 532 articles from the initial 621 articles were excluded after screening, based on in-depth observations of the abstract content of the articles. The second screening yielded 89 articles that met the criteria. Of the 89 articles, additional in-depth observations of the full text of all the articles were made. A total of 46 articles remained after this stage 3 screening. Upon completion of this final stage, a total of 38 publications and 5 additional articles through manual reference tracing were included in the final data collection and further analyzed. All stages of systematic screening of the articles are shown in Figure 6.

### 3.3. Data Extraction

During the final selection, data were drawn from 43 studies on fucosterol. The data collection process was based on two main themes: (1) the characteristics of the articles, and (2) the criteria used to determine the bioactivity, safety, and toxicity of fucosterol. Specifically, the following features were extracted: year of publication, name of journal, geographic area where the research was conducted, author affiliation, publisher, biological activity of fucosterol, source of fucosterol, method used, experimental model, concentration of fucosterol, safety and toxicity of fucosterol.

## 4. Conclusions

Our review revealed that fucosterols derived from macroalgae can potentially be applied in the nutraceutical and pharmacological industries based on its bioactivities, safety, and toxicity. Fucosterol derived from brown macroalgae exhibits potentially beneficial activities; however, certain research gaps should be addressed. Despite the uncovered biological activities, most studies remain at a preliminary stage, and some of them have not included the study of safety and toxicity to organisms; hence, more in-depth studies are required. The studies of bioactivity, safety, and toxicity of fucosterol have been carried out mostly at the in vitro level (52%), and only 26% have been conducted at the in vivo level using a mouse model. An in vivo experiment can provide fundamental data for understanding the mechanism of nutraceutical and pharmacological properties of sterol before it is used in clinical trials. However, we did not find any clinical studies on fucosterol published between 2002 and 2020. Studies on the safety and toxicity levels of fucosterol at the clinical stage are very important for the development of this sterol for the nutraceuticals and pharmaceutical industry. Brown macroalgae (Phaeophyta) are a major source of fucosterol. A total of 2071 species belonging to the class Phaeophyta have been recorded [68]. However, the study of fucosterol in brown macroalgae continues to be limited to only certain species. *Hizikia fusiformis/Sargassum fusiforme* and *Ecklonia cava* subsp. *stolonifera* are the two species with the highest number of publications. Additional studies on other Phaeophyta species are required to elucidate the fucosterol content in specific algal classes. Future comprehensive research on fucosterol, including the study of macroalgae sources, chemical characterization, pharmacokinetic mechanisms, in vitro, in vivo, and clinical experiments will elucidate the role of fucosterol as a potent bioactive compound derived from marine sources.

## Figures and Tables

**Table 1:** Studies on safety and toxicity of fucosterol extracted from macroalgae, tested in bacteria and fungi.

Bacteria/ Fungi	Extract or Chemical	Sources	Method	Concentration	Toxicity	Ref.
*Pyricularia oryzae*	Fucosterol extract	*Sargassum* *carpophyllum*	Screening	Nd	Affected the morphology	[22]
*Staphylococcus* *epidermidis*	Fucosterol extract	*Turbinaria conoides*	Broth dilution susceptibility assay	2–256 µg/mL	Inhibited bacteria growth	[58]
*Aspergillus niger*	Fucosterol extract	*Turbinaria conoides*	Broth dilution susceptibility assay	2–256 µg/mL	Inhibited fungal growth	[58]
*Candida albicans*	Fucosterol extract	*Turbinaria conoides*	Broth dilution susceptibility assay	2–256 µg/mL	Inhibited fungal growth	[58]
*Escherichia coli*	Fucosterol extract	*Turbinaria conoides*	Broth dilution susceptibility assay	2–256 µg/mL	Inhibited bacteria growth	[58]
*Staphylococcus* *aureus*	Fucosterol extract	*Turbinaria conoides*	Broth dilution susceptibility assay	2–256 µg/mL	Inhibited bacteria growth	[58]
*Pseudomonas* *aeruginosa*	Fucosterol extract	*Turbinaria conoides*	Broth dilution susceptibility assay	2–256 µg/mL	Inhibited bacteria growth	[58]
*Fusarium* *culmorum*	Commercial fucosterol	Nd	Determined on a liquid RB medium	0.05–1.0%	Inhibited fungal growth and caused total degradation	[57]

Nd: not determined.

**Table 2:** Studies on safety and toxicity of fucosterol extracted from macroalgae, tested in human and animal cell lines.

Cell Lines	Extract or Chemical	Sources	Method	Concentration	Toxicity	Ref.
RAW 264.7 macrophage cells	Fucosterol extract	*Padina boryana*	MMT assay and incubated for 23 h	12.5, 25, and 50 µg/mL	Increased cell viability	[43]
A549 human lung epithelial cells exposed to CPM	Fucosterol extract	*Sargassum aquifolium* (formerly *Sargassum binderi*)	MMT assay and incubated for 24 h	3.125, 6.25, 12.5, 25, 50, and 100 µg/mL	Low toxicity and increased cell viability	[44]
Chinese hamster ovary (CHO) cells, rat basophil leukemia (RBL) cells, U373 cells, and BA/F3 cells	Fucosterol extract	*Undaria pinnatifida* and *Eisenia bicyclis*	Human monoamine oxidase (*h*MAO) inhibition and functional assay	500 μM	Had no effects on the viability of the cells	[55]
Human dermal fibroblasts (HDF) and HaCaT cells	Fucosterol extract	*Sargassum aquifolium* (formerly *Sargassum binderi*)	MMT assay and incubated for 3 h	3.125, 6.25, 12.5, 25, 50, and 100 µg/mL	Not toxic and increased cell viability	[60]
Human recombinant PTP1B	Fucosterol extract	*Sargassum fusiforme*	Docking simulation	0 to 2 mM	Inhibited PTP1B and α-glucosidase	[28]
RAW 264.7 macrophages	Fucosterol extract	*Undaria pinnatifida*	Western blot analysis	10, 25, or 50 µM	Had no effects on the viability of the cells	[42]
Murine 3T3-L1 preadipocytes	Fucosterol extract	*Ecklonia cava* subsp. *stolonifera* (formerly *Ecklonia stolonifera*)	Western blot analysis	25 and 50 µM	No significant effects up to 50 µM	[15]
β-Site amyloid precursor protein cleaving enzyme 1 (BACE1)	Fucosterol extract	*Undaria pinnatifida* and *Ecklonia cava* subsp. *stolonifera* (formerly *Ecklonia stolonifera*)	Kinetics and molecular docking simulation	0, 5.0, 20, and 100 µM	Inhibited BACE1 and not toxic	[53]
Insulin-resistant HepG2 (human hepatocarcinoma) cells	Fucosterol extract	*Ecklonia cava* subsp. *stolonifera* (formerly *Ecklonia stolonifera*)	MMT assay and incubated for 2 h	12.5, 25, 50, 100, and 200 µM	No significant effect up to 100 µM	[27]
HepG2 cells induced t-BHP and tacrine	Fucosterol extract	*Ecklonia cava* subsp. *stolonifera* (formerly *Ecklonia stolonifera*)and *Eisenia bicyclis*	MMT assay and incubated for 2 h	0, 25, 50 and 100 μM	Low toxicity and increased cell viability	[47]
HaCaT cells induced cobalt chloride (CoCl_2_)	Fucosterol extract	*Sargassum fusiforme* (formerly *Hizikia* *fusiformis*)	MMT assay and incubated for 2 h	1, 2, 5, and 10 μM	Low toxicity and increased cell viability	[61]
C8-B4 microglial cells	Fucosterol extract	*Padina australis*	MMT assay and incubated for 4 h	12, 24, 48, 96, and 192 μM	Had no effects on the viability of the cells	[54]
Colon carcinoma (HT-29), colorectal adenocarcinoma (Caco-2), and breast ductal carcinoma (T47D) cell lines	Fucosterol extract	*Sargassum angustifolium*	MMT assay and incubated for 4 h	4.5, 18, 36, and 72 μg/mL	Low toxicity	[38]
Oral carcinoma (KB), epithelial carcinoma of the larynx (Hep-2), MCF-7, cervix adenocarcinoma (SiHa), and a human cell embryonic kidney cell line (HEK-293)	Fucosterol extract	*Dictyota ciliolataPadina sanctae-crucis,* and *Turbinaria tricostata*	MMT assay and incubated for 2 h	Nd	Only inactive on HEK-293	[37]
Dalton’s Lymphoma Ascites (DLA) cells	Fucosterol extract	*Turbinaria conoides*	Trypan blue viability assay	100 and 200 μg/mL	Not toxic	[36]
Lung cancer cell and human normal cell	Commercial fucosterol	Nd	MMT assay and incubated for 24 h	1.55, 3.12, 6.25, 12.5, 25, 50, and 100 µg/mL	Decreased cell viability in cancer cell and low toxicity in normal cell	[17]
Human cancer cell lines (HT29 and HCT116) and CCD-18Co fibroblasts	Commercial fucosterol	Nd	MMT assay and incubated for 24 h	5 and 10 µM	Decreased cell viability in HT29 cells, but no effect in HCT116 and CCD-18Co	[62]
Human promyelocytic leukemia cells (HL-60)	Commercial fucosterol	Nd	MMT assay and incubated for 4 h	7.55, 15.1, 30.2, 60.4, and 120.8 µM	Not toxic	[63]
Human ovarian cancer (ES2 and OV90) cells	Commercial fucosterol	Nd	2′,7′-dichlorofluorescin diacetate assay	0, 20, 40, 60, 80, and 100 µM	Not toxic	[35]
HaCaT cells and monkey kidney COS-7 cells	Commercial fucosterol	Nd	MMT assay and incubated for 3 h	0.5, 1, and 5 μM	Had no effects on the viability of the cells	[45]

Nd: not determined.

**Table 3:** Studies on safety and toxicity of fucosterol extracted from macroalgae, tested in animals.

Animal	Extract or Chemical	Sources	Method	Concentration	Toxicity	Ref.
E18 aging rats	Fucosterol extract	*Ecklonia cava* subsp. *stolonifera*	Dorsal hippocampus injected by fucosterol for 4 weeks	10 µmol/h	Increased the latency to reach the platform	[52]
C57BL/6 mice	Fucosterol extract	*Sargassum* *fusiforme*	Oral administration	50, 100, and 200 mg/kg	Increased splenocyte proliferation and increased NO production with no cytotoxicity	[34]
Balb/e mice	Fucosterol extract	*Sargassum* *fusiforme*	Administered by via gastric intubation route	0.1 mL/20 g of mouse	Not neurotoxic	[66]
Ovariectomized (OVX) rats	Fucosterol extract	*Sargassum* *fusiforme*	Oral, for 7 weeks beginning 12 weeks post-operation	25, 50, and 100 mg/kg	Had no toxic effects	[31]
*Caenorhabditis elegans*	Fucosterol extract	*Sargassum* *fusiforme*	Measured on both NGM agar and broth containing fucosterol	Up to 0.1 mg/mL in 2% dimethyl sulfoxide (DMSO)	Low toxicity	[56]
Institute of Cancer Research (ICR) mice	Fucosterol extract	*Ecklonia cava* subsp. *stolonifera* and *Eisenia* *bicyclis*	Oral, for 3 consecutive days	200 μL fucosterol (25, 50, and 100 mg/kg)	No mortality	[47]
BALB/c mice weighing	Commercial fucosterol	Nd	Oral, administered daily for 3 days	25, 50, or 100 mg/kg	Inhibited ConA-induced acute liver injury significantly	[50]

## Data Availability

Data supporting reported results are available upon request.

## Abbreviations

5-Fu, 5-fluorouracil; AchE, acetylcholinesterase; ALT, alanine aminotransferase; AR, aldose reductase; RLAR, rat lens aldose reductase; AST, aspartate aminotransferase; BACE1, β-secretase 1; BchE, butyrylcholinesterase; Bcl-2, B-cell lymphoma-2; ConA, concanavalin A; COX-2, cyclooxygenase-2; ERK, extracellular-signal-regulated kinase; EtOAc, ethyl acetate; FoxO, forkhead box protein O; GRP78, glucose regulatory protein 78; GSH, glutathione; GSH-px, glutathione peroxidase; HAB, harmful algal blooms; HDL, high density lipoprotein; HO-1, heme oxygenase-1; HRAR, human recombinant aldose reductase;IC_50,_ half-maximal inhibitory concentration; IL-6, interleukin-6; IL-1β, interleukin-1β; iNOS, inducible nitric oxide synthase; LDL, low density lipoprotein; LPS, lipopolysaccharide; LXR, liver X receptor; MAO, monoamine oxidase; MAPK, microtubule associated protein kinase; MEK, mitogen-activated protein kinase; MIC, minimum inhibitory concentration; MMP-1, matrix metalloproteinase-1; MMPs, matrix metalloproteinases; mRNA, messenger RNA; MTT, (3-(4, 5-dimethyl thiazole-2-yl)-2, 5-diphenyltetrazolium bromide); mTOR, mammalian target of rapamycin; NF-κB, nuclear factor-κB; NGM, nematode growth medium; NO, nitric oxide; Nrf2, nuclear factor erythroid 2-related factor 2; P53, protein 53; p-c-Fos, phospho-c-Fos; p-c-Jun, phospho-c-Jun; PI3K/Akt, phosphatidylinositol 3-kinase/protein kinase B; PM, particulate matter; PPAR, proteins peroxisome proliferator-activated receptor; PPARγ, peroxisome proliferator- activated receptor gamma; PTP1B, protein tyrosine phosphatase 1B; Raf, mitogen-activated protein kinase; ROS, reactive oxygen species; sGOT, serum glutamic oxaloacetic transaminase; sGPT, serum glutamic pyruvic transaminase; SIRT1, sirtuin 1; SOD, superoxide dismutase; t-BHP, tert-butyl hydroperoxide; TGF-1, transforming growth factor-1; TLC, thin layer chromatography; TNF-α, tumor necrosis factor alpha; UVB, ultraviolet B.
